# Knowledge and skills on active management of third stage of labor for prevention of post-partum haemorrhage among health care providers in Lake Zone, Tanzania: a cross sectional study

**DOI:** 10.1186/s12905-022-01616-1

**Published:** 2022-02-11

**Authors:** Daniel Lugwesa Muyanga, Angelina A. Joho

**Affiliations:** 1 Department of Obstetrics and Gynecology, Bugando Medical Center, Mwanza, Tanzania; 2 Department of Clinical Nursing, School of Nursing and Public Health, The University of Dodoma, Dodoma, Tanzania

**Keywords:** Knowledge, Skills, AMTSL, Health care providers

## Abstract

**Background:**

Health care providers (HCPs) knowledge and skills are both crucial in saving the lives of mothers and their newborns during childbirth. This study aimed to assess the knowledge and skills of HCPs on active management of third stage of labor (AMTSL) for prevention of PPH in Lake Zone Tanzania.

**Methods:**

A cross-sectional analytical hospital-based study which included 340 HCPs who were randomly selected, the study was conducted from March to May 2019 in lake zone, Tanzania. Data were collected using standardized questionnaire and observational checklist. Predictors of knowledge and skills on AMTSL were determined using binary logistic regression under multivariable analysis using SPSS version 23.0. p-value less than 0.05 was considered significant.

**Results:**

Most 200 (58.8%) of the participants were aged between 25 and 34 years with mean age 31.4 ± 6.26 years. Majority 240 (67.6%) were females. Of all HCPs, 171 (50.3%) had adequate knowledge whereas 153 (45.0%) had adequate skills on AMTSL. Males (AOR = 1.96, 95% CI 1.18–3.26), HCPs with University education (AOR = 3.29, 95% CI 1.19–9.13) and previous BEmONC training (AOR = 2.20, 95% CI 1.24–3.91) were found to be the predictors of adequate knowledge on AMTSL. HCPs aged ≥ 45 years (AOR = 9.35, 95% CI 1.74–10.28) and HCPs working at a hospital (AOR = 1.78, 95% CI 1.12–2.82) were associated with having adequate skills on AMTSL.

**Conclusion:**

HCPs included in this study demonstrated low skills on AMTSL as compared to knowledge which needs immediate attention. We recommend continuous in-service training and supportive supervision among HCPs working in labour wards for improving their knowledge and skills on AMTSL. This will help to reduce maternal morbidity and mortality related to PPH.

## Background

In 2015, maternal mortality rate has been estimated to be 216 per 100,000 live births globally [1–3]. Across regions estimate, the overall MMR in developing regions is 239 per 100,000 live births which is approximately 20 times higher than that in developed regions where the estimation is 12 per 100,000 live births [1].

In Tanzania, MMR was indicated to be 556 per 100,000 live births in 2016 [4, 5]. The major direct causes of maternal deaths are obstetric hemorrhage, hypertensive disorders in pregnancy, sepsis, abortion complications and obstructed labor [6, 7]. However, PPH is the leading cause of maternal death with approximately 25% of all deaths [6]. Uterine atony is amongst the leading cause of PPH as a result of failure of the uterus to contract after delivery. Other causes of PPH include trauma following rupture of the uterus, vaginal or cervical laceration, retained tissues and bleeding disorders [8].

PPH can be prevented by effectively use of AMTSL including timely uterotonic administration within 1 min after ruling out of absence of second baby, delivery of placenta using controlled cord traction and uterine massage every 15 min for the first 2 hours [8, 9].

The Tanzanian government has committed herself on preventing PPH using different measures including training HCPs working in labor wards in Basic Emergency Obstetric and Neonatal Care (BEmONC) and Comprehensive Emergency Obstetric and Neonatal Care (CEmONC) including AMTSL [6].

Dispite the Tanzanian government efforts, maternal dealths caused by PPH are still higher [10]. Moreover, researchers have reported that utilization of AMTSL is very low in African countries in which Tanzania is included with estimate of 0.5–17.6% utilization of AMTSL [11]. HCPs knowledge and skills are pre requisite in preventing maternal deaths caused by PPH.

Therefore, this study aimed to determine knowledge and skills among HCPs on AMTSL in Lake Zone, Tanzania.

## Methods

### Study area and design

This was analytical cross-sectional study using quantitative approach. The study assessed HCPs knowledge and skills on AMTSL which was conducted from March to May 2019 in Lake Zone, in Tanzania mainland. Lake zone is located in the North-Western part of Tanzania with a population of 13,662,883 of whom 3,103,563 were women of child bearing age (15–49 years) (Lake zone RCH report 2017). The zone has six regions including Kagera, Mwanza, Shinyanga, Mara, Geita and Simiyu. For the case of this study, two regions (Mwanza and Geita) were randomly selected.

### Study population

The study included HCPs working in labor ward who were conducting spontaneous vaginal delivery in different level of health facilities selected from two regions of Lake Zone. Those who were off duty, on leave and sick were excluded to participate.

### Sampling procedure and sample size

Simple random sampling by lottery replacement method was used in selecting the two regions and districts, respectively. We prepared six pieces of paper representing six region names from the lake zone. The papers were folded and put in a small box and vigorously shaken in order to mix them. Then research assistant picked randomly two out of the six papers randomly representing the selected regions. Two regional referral hospitals from selected regions were selected conveniently and two district hospitals from each selected region were selected randomly. Two health centers from each selected district were selected by using simple random sampling. After our review of HCPs records in the selected hospitals in a year, we found out that there were 670, 552, 310, 205, 45, and 56 of HCPs. Proportionate sampling technique was used to obtain the required number of participants from each of the selected hospitals. ni = (Ni/Nt) x n [12]. Where ni = required number of study participants from a given hospital, Ni = required sample size for the study, Nt = total number of HCPs from all the selected hospitals, and n = number of HCPs as per hospital. A total number of 1838 HCPs were available in all selected hospitals, the sample size of 350 were proportionately allocated in the six selected hospitals thus 127, 105, 59, 39, 9, and 11 study participants were selected. Convenient sampling method was used to select the study participants.

### Data collection procedures and tools

Data were collected using a self-administered structured questionnaire and an observational checklist. The questionnaire was divided into two sections. The first section explored socio-demographic characteristics of participants and the second section contained questions for assessing knowledge of HCPs on AMTSL as it was done in the previous study [13]. The observational checklist was used for assessing skills of HCPs [13, 14]. Three nurse midwives were used as research assistants, who had attended a two days training regarding research ethics and orientated on  the data collection tools. The research assistants observed all the HCPs as they were conducting deliveries in the labor wards this included observing all the key points of care related to AMTSL.

The questionnaire was administered to the participants in a private room within the labor ward which took an average of 25–30 min to complete. The research assistants were introduced to HCPs by the in charge of the labor ward. Participation was voluntary and the questionnaire was anonymously filled and participants had an opportunity to withdraw from the study at any point without any penalty.

### Measurement of variables

#### Level of knowledge and skills

AMTSL for prevention of PPH among HCPs was measured using a structured questionnaire. Knowledge questions contained 10 items of which the right answer scored one point and the wrong answer had a zero score [13]. Participants who scored five points and above were considered as having adequate knowledge on AMTSL and those who scored less than five were termed as having inadequate knowledge on AMTSL. On the other hand, the level of skills on AMTSL was measured using observational checklist. The checklist contained 10 items which were observed while conducting the deliveries. Each item of a correct performance scored one point, and zero point for incorrect score. HCPs who scored more than 5 points were regarded that had adequate skills. We adapted the Prevention of Postpartum Hemorrhage Initiative national survey (POPPHI-2006) which was used by Bishanga et al. [13].

### Data analysis

Statistical package for the social sciences (SPSS) program version 23.0 was used to analysis data collected. Descriptive statistics was used to analyze demographic characteristics and results were presented in proportions. Pearson Chi-square statistical test was used to assess the association between categorical variables. Inferential statistics comprised of logistic regression analysis for determining of predictors of knowledge and skills regarding AMTSL. Determination of the predictors of knowledge and skills regarding AMTSL was done by adjusting for all variables and p < 0.05 was considered significant.

## Results

### Socio-demographic characteristics

There were 1838 HCPs working in the labor ward from all six health facilities selected. Among them, 350 (19%) HCPs were selected to participate in the study. Those who participated in the study were 340 while 10 participants dropped out from the study for various reasons, this corresponds to a 97% response rate.

Most of participants 200 (58.8%) were aged 25–34 years with mean age of 31.4 ± 6.26 years. Majority 240 (67.6%) were females. Regarding professional education, most of the HCPs 182 (53.5%) had diploma. Majority 297 (87.4%) of the HCPs had five years of working experience in labor ward whereas 262 (77.1%) had five years working experience in obstetric unit. Over half of the HCPs 172 (50.6%) had training in AMTSL, BemONC and CemONC (Table 1).

**Table 1:** Distribution of socio-demographic characteristics of the study respondents (N = 340)

Variables	Frequency (n)	Percentage (%)
Age (years)		
< 25	56	16.5
25–34	200	58.8
35–44	68	20
≥ 45	16	4.7
Sex		
Female	230	67.6
Male	110	32.4
Marital status		
Single	128	37.6
Married	212	62.4
Level of education		
Certificate	112	32.9
Diploma	182	53.5
Degree	46	13.5
Professional qualification		
AMO/MD	36	10.6
Registered nurse	192	56.5
Enrolled nurse	112	32.9
Working experience in obstetric unit (years)		
< 5	262	77.1
≥ 5	78	22.9
Working experience in LW (years)		
< 5	297	87.4
≥ 5	43	12.6
Type of facility		
Health centre	168	49.4
Hospital	172	50.6
Past training on obstetric and newborn emergencies		
Not trained	172	50.6
AMTSL	39	11.5
BemONC	92	27.1
CEmONC	15	4.4
Both (AMTSL, BemONC, CEmONC)	22	6.5
Time of training on obstetric and newborn emergencies		
Pre-service	178	52.4
In-Service	105	30.9
Pre-service & in-service	57	16.8

AMO—Assistant medical officer, MD—medical doctor, AMTSL—Active management of third stage of labor, BemONC—Basic emergency obstetric and newborn care, CEmONC—Comprehensive emergency obstetric and newborn care

### Knowledge and skills on AMTSL among HCPs

**Fig. 1:**
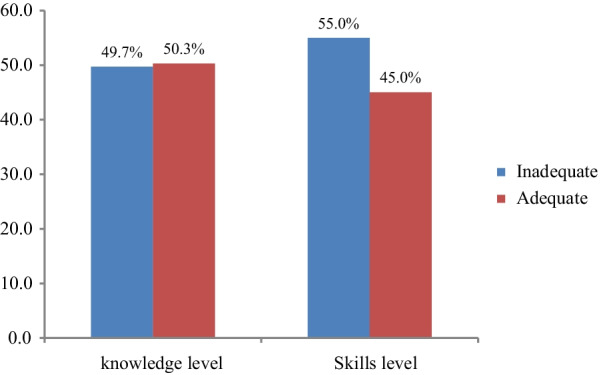
Knowledge and skills o AMTSL among health care providers

Out of 340 HCPs, 171 (50.3%) and 145 (45%) had adequate knowledge and skills on AMTSL, respectively (Fig. 1).

### Item analysis of skills on AMTSL among HCPs

A total of 10 items were analysed in assessing skills of HCPs on AMTSL. Results revealed that, 335 (98.5%) of them were performing correctly in item number 1 (correct amount of uterotonic drugs administered). Additionally, only 115 (33.8%) performed correctly in item number 10 (i) (timely uterotonic administration within one minute in the absence of a second baby). Contrary, majority of the HCPs 297 (87.4%) performed correctly in item number 4 (performing controlled cord traction with counter-traction) whereas 134 (39.4%) performed correctly in item number six (uterine massage every 15 min for first 2 h) (Table 2).

**Table 2:** Skills related to active management of third stage of labor (N = 340)

Item	Correct performance	Incorrect performance
	n (%)	n (%)
1. Correct amount of uterotonic drugs administered after assessing absent of second baby	335 (98.5)	5 (1.5)
2. Massage of uterus while placenta is inside	200 (58.8)	140 (41.2)
3. Counter traction done by next contraction	157 (46.2)	183 (53.8)
4. Performing CCT with counter-traction	297 (87.4)	43 (12.6)
5. Immediate uterine massage	252 (74.1)	88 (25.9)
6. Uterine massage every 15 min for first 2 h	134 (39.4)	206 (60.6)
7. Placenta and membrane examination before dispose	217 (63.8)	123 (36.2)
8. Perennial, vaginal, placenta and membrane assessment	320 (94.1)	20 (5.9)
9. Technique of placental delivery	269 (79.1)	71 (20.9)
10. Timely uterotonic administration		
(i) Within one minute	115 (33.8)	
(ii) One to three minutes	173 (50.9)	
(iii) More than Three minutes	52 (15.3)	

### Association between Socio-demographic factors and level of knowledge on AMTSL

Results from Chi-square indicated that, sex of HCPs, age, education level, professional qualification, working experience in obstetric unit and labour ward, past training in BemONC, CemONC, AMTSL and duration of training were statistically significant (p < 0.05) (Table 3).

**Table 3:** Association between socio- demographic factors and level of knowledge on AMTSL (N = 340)

Demographic characteristics	Level of knowledge		Chi-square	P-value
	Inadequate n (%)	Adequate n (%)		
Age (years)				
< 25	37 (66.1)	19 (33.9)		
25–34	98 (49.0)	102 (51.0)		
35–44	28 (41.2)	40 (58.8)		
≥ 45	6 (47.5)	10 (62.5)	8.972	0.03
Sex				
Female	126 (54.8)	104 (45.2)		
Male	43 (49.1)	67 (60.9)	7.329	0.007
Marital status				
Single	72 (56.3)	56 (43.8)		
Married	97 (45.8)	115 (54.2)	3.517	0.061
Education level				
Certificate	65 (58.0)	47 (42.0)		
Diploma	89 (48.9)	93 (51.1)		
Degree	15 (32.6)	31 (67.4)	8.535	0.014
Professional qualification				
AMO/MD	10 (27.8)	26 (72.2)		
Registered nurse	94 (49.0)	98 (51.0)		
Enrolled nurse	65 (58.0)	47 (42.0)	10.076	0.006
Years working in obstetric				
< 5	141 (53.8)	121 (46.2)		
≥ 5	28 (35.9)	50 (64.1)	7.720	0.005
Years at working in LW				
< 5	155 (52.2)	142 (47.8)		
≥ 5	14 (32.6)	29 (67.4)	5.790	0.016
Facility level				
Health centre	80 (47.6)	88 (52.4)		
Hospital	89 (51.7)	83 (48.3)	0.579	0.447
Past training on obstetric and newborn emergencies				
Not trained	103 (59.9)	69 (40.1)		
AMTSL	19 (48.7)	20 (51.3)		
BemONC	33 (35.9)	59 (64.1)		
CEmONC	8 (53.3)	7 (46.7)		
Both (AMTSL, BemONC, CEmONC)	6 (27.3)	16 (72.7)	18.695	0.001
Time of training on obstetric and newborn emergencies				
Pre-service	108 (60.7)	70 (39.3)		
In-Service	44 (41.9)	61 (58.1)		
Pre-service & in-service	17 (29.8)	40 (70.2)	20.134	0.006

AMO—Assistant medical officer, MD—medical doctor, AMTSL—Active management of third stage of labor, BemONC—Basic emergency obstetric and newborn care, CEmONC—Comprehensive emergency obstetric and newborn care

### Predictors on level of knowledge on AMTSL

After adjusting for all factors, males HCPs were found be almost 2 times more likely to have adequate knowledge on AMTSL compared to female (AOR = 1.96, 95% CI 1.18–3.26, p < 0.05). HCPs with certificate level of education were 0.82 less likely to have adequate knowledge regarding AMTSL compared to those with degree level of education and those with diploma level of education were 0.29 less likely to have adequate knowledge regarding AMTSL compared to those with degree level of education (AOR = 0.82, 95% CI 0.49–6.89, p = 0.379, AOR = 0.29, 95% CI 1.19–9.13, p = 0.022, respectively). Registered and enrolled nurses were less likely 0.56, 0.30 to have adequate knowledge compared with AMO/MD (AOR = 0.56, 95% CI 0.19–1.76, p = 0.331, AOR = 0.30, 95% CI 0.12–0.73, p = 0.008, respectively). Those with previous BEmONC training were 2 times more likely to have adequate knowledge compared to those who did not receive training (AOR = 2.20, 95% CI 1.24–3.91, p < 0.05). Age, marital status and type of health facility were not associated with level of knowledge on AMTSL (p > 0.05) (Table 4).

**Table 4:** Predictors of knowledge level on AMTSL (N = 340)

Variable	COR	95% C. I		p value	AOR	95% C. I		p value
		Lower	Upper			Lower	Upper	
Age (years)								
< 25	2.03	1.09	3.76	0.025	1.14	0.54	2.40	0.737
25–34	2.78	1.34	5.80	0.006	1.29	0.37	2.74	0.062
35–44	3.25	1.02	10.29	0.045	0.93	0.23	3.82	0.923
**˃** 45	(ref)							
Sex								
Female	(ref)							
Male	1.89	1.19	3.0	0.007	1.96	1.18	3.26	0.009
Marital status								
Single	1.52	0.97	2.38	0.061	1.33	0.77	2.34	0.316
Married	(ref)							
Education level								
Certificate	1.45	0.90	2.32	0.128	0.82	0.49	6.89	0.379
Diploma	2.86	1.39	5.88	0.004	0.29	1.19	9.13	0.022
Degree	(ref)							
Prof. qualification								
AMO/MD	(ref)							
Registered nurse	0.40	0.18	0.88	0.022	0.56	0.19	1.76	0.331
Enrolled	0.28	0.12	0.63	0.002	0.30	0.12	0.73	0.008
Health facility								
Health center	0.85	0.55	1.30	0.447	0.78	0.49	1.24	0.299
Hospital	(ref)							
Previous training								
Not trained	1.57	0.78	3.16	0.204	0.19	0.56	2.50	0.665
AMTSL	2.67	1.57	4.51	0.005	2.20	1.24	3.92	0.007
BemONC	1.31	0.45	3.77	0.621	0.64	0.20	2.09	0.461
CEmONC	3.98	1.48	10.68	0.006	1.78	0.56	5.69	0.336
Both (AMTSL, BemONC, CEmONC)	(ref)							
Working in obstetric units (years)								
< 5	2.08	1.23	3.51	0.006	1.56	0.71	3.47	0.271
≥ 5	(ref)							
Experience in labor ward (years)								
< 5	2.26	1.15	4.45	0.018	1.49	0.58	3.80	0.408
≥ 5	(ref)							

AMO—Assistant medical officer, MD—medical doctor, AMTSL—Active management of third stage of labor, BemONC—Basic emergency obstetric and newborn care, CEmONC—Comprehensive emergency obstetric and newborn care

### Association between Socio-demographic factors and level of skills on AMTSL

Findings from Chi-square indicated that, age (x^2^ = 16.63, p < 0.05), marital status (x^2^ = 4.67, p < 0.05), facility level (x^2^ = 6.40, p < 0.05), past training in BemONC, CemONC, AMTSL (x^2^ = 14.06, p < 0.05) and time of training (x^2^ = 10.98, p < 0.05) had association with skills level on AMTSL among HCPs (Table 5).

**Table 5:** Association between socio-demographic factors and level of knowledge on AMTSL (N = 340)

Variables	Skills		Chi-square	p-value
	Inadequate (%)	Adequate (%)		
Age (years)				
< 25	39 (69.6)	17 (30.4)		
25–34	108 (54,0)	92 (46.0)		
35–44	38 (55.9)	30 (44.1)		
≥ 45	2 (12.5)	14 (87.5)	16.630^a^	0.001
Sex				
Female	120 (52.2)	110 (47.8)		
Male	67 (60.9)	43 (39.1)	2.294^a^	0.13
Age (years)				
< 25	39 (69.6)	17 (30.4)		
25–34	108 (54,0)	92 (46.0)		
35–44	38 (55.9)	30 (44.1)		
≥ 45	2 (12.5)	14 (87.5)	16.630^a^	0.001
Marital status				
Single	80 (62.5)	48 (37.5)		
Married	107 (50.5)	105 (49.5)	4.666^a^	0.031
Education level				
Certificate	55 (49.1)	57 (50.9)		
Diploma	106 (58.2)	76 (41.8)		
Degree	269 (56.5)	20 (43.5)	2.387^a^	0.303
Professional qualification				
AMO/MD	19 (52.8)	17 (47.2)		
Registered nurse	113 (58.9)	79 (41.1)		
Enrolled nurse	55 (49.1)	57 (50.9)	2.796^a^	0.247
Worked experience in obstetric unit (years)				
Less than 5 years	146 (55.7)	116 (44.3)		
5 years and above	41 (52.6)	37 (47.4)	0.243^a^	0.622
Working experience in facility (years)				
Less than 5	165 (55.6)	132 (44.4)		
5 and above	22 (51.2)	21 (48.8)	0.293^a^	0.588
Facility level				
Health centre	104 (61.9)	64 (38.1)		
Hospital	83 (48.3)	89 (51.7)	6.397^a^	0.011
Previous training				
Not trained	109 (63.4)	63 (36.6)		
AMTSL	15 (38.5)	24 (61.5)		
BemONC	41 (44.6)	51 (55.4)		
CEmONC	10 (66.7)	5 (33.3)	14.055^a^	0.007
Both (AMTSL, BemONC, CemONC)	12 (54.5)	10 (45.5)		
Time of training				
Pre-service	113 (63.5)	65 (36.5)		
In-service	49 (46.7)	56 (53.3)		
Pre-service & in-service	25 (43.9)	32 (56.1)	10.980^a^	0.004

### Association between socio-demographic factors and level of skills on AMTSL

After adjusting for all factors HCPs aged ≥ 45 years were 9 times likely to have adequate skills on AMTSL compared with those aged below 25 years (AOR = 9.35, 95% CI 1.74–10.28, p < 0.05). HCPs who were working at a hospital were almost twofold more likely to have adequate skills than those who works at health centres (AOR = 1.78, 95% CI 1.12–2.82, p < 0.05). Marital status, previous training and time of training were not statistically significant (Table 6).

**Table 6:** Association between socio-demographic factors and level of skills on AMTSL among HCPs (N = 340)

Variables	OR	p value	95% CI		p value	AOR	95% CI	
			Lower	Upper			Lower	Upper
Age (years)								
< 25	ref							
25–34	1.95	0.038	1.04	3.68	0.372	1.39	0.67	2.89
35–44	1.81	0.118	0.86	3.81	0.789	1.13	0.46	2.81
≥ 45	16.06	0.001	3.28	78.54	0.009	9.35	1.74	50.28
Marital status								
Single	1.64	0.031	1.05	2.56	0.459	1.23	0.71	2.12
Married	ref							
Facility level								
Health centre	1.74	0.012	1.13	2.68	0.014	1.78	1.12	2.82
Hospital	ref							
Previous training								
Not trained	2.77	0.005	1.35	5.66	0.534	1.85	0.27	12.93
AMTSL	2.15	0.004	1.29	3.60	0.743	1.38	0.20	9.48
BemONC	0.87	0.799	0.28	2.65	0.450	0.48	0.07	3.22
CEmONC	1.44	0.423	0.59	3.53	0.688	0.66	0.09	4.98
Both (AMTSL, BemONC, CemONC)	ref							
Time of training								
Pre-service	1.99	0.006	1.22	3.24	0.902	1.13	0.17	7.58
In-service	2.23	0.01	1.21	4.08	0.520	1.86	0.28	12.23
Pre-service & in-service	ref							

## Discussion

Knowledge and skills related to active management of third stage of labor is of paramount importance for the prevention of PPH. In the current study, there was a mismatch between knowledge and skills on AMTSL among HCPs working in labor wards. The key findings in the current study include positive association between HCPs’ adequate knowledge on AMTSL and being male HCP, being AMO/MD, having studied to degree level in nursing and medicine and being exposed to previous training on BeMOC. Age of HCPs above 45 years and working in hospitals were positively associated with skills related to AMTSL.

Male health care providers were positively associated with adequate knowledge on AMTSL. This is contrary to finding from study done by Lami and Deksisa reported that being female was less associated with knowledge of AMTSL (AOR = 3.83, 95% CI 1.39–10.57) [11]. HCPs in our study who were having degree in nursing and medicine had almost twofold higher odds of adequate knowledge in AMTSL compared to women. This is contrary to what was reported in a study of Adane et al., in which male health care providers were associated with good practice (AOR = 1.74, 95% CI 1.03–2.94) [1].

Regarding professional qualification, enrolled and registered nurses were less likely to have adequate knowledge of AMTSL compared to AMO/MD. This could be due to a number of reasons such as long period of training for medical doctors, being in field for more than five years and in-service training on AMTSL which might helped them of having adequate knowledge [4, 11, 15]. However, the training for enrolled nurses in Tanzania is two years, of which is very short for them to be equipped with knowledge related to obstetric emergency prevention which include AMTSL.

In the current study, health care providers who had higher education level (degree) in nursing and medicine were positively associated with knowledge of AMTSL. This is in line with study conducted in Ethiopia by Henok et al., in which they reported that health care providers with higher education were having adequate knowledge of AMTSL (AOR = 8.51, 95% CI 2.02–35.95) [16].

Health care providers who had previous training on BeMOC and AMTSL were significantly associated with knowledge of AMTSL. This has also been reported elsewhere. For instance, in a study conducted in Ethiopia by Henok reported that health care providers who had on job training on AMTSL were more knowledgeable compared to their counterparts (AOR = 3.13, 95% CI 1.0–9.8) [16]. Another study in Ethiopia reported pre and/or in-service training was associated with HCPs knowledge [17]. Also, another study conducted in Ethiopia reported that those who had in-service training in prevention of postpartum haemorrhage using AMTSL were knowledgeable (AOR = 2:12, 95% CI 1.14–3.39) [15].

Level of skills among HCPs was another aspect of AMTSL that was assessed in the current study. We observed that, majority of HCPs had inadequate skills on AMTSL for prevention of postpartum haemorrhage. A few HCPs demonstrated adequate skills on AMTSL including providing correct dose of uterotonic drugs, timely administration of uterotonic drugs within one minute and controlled cord traction and massaging the uterus after every 15 min within two hours of delivery similar to the studies reported elsewhere [11, 16, 17].

However, in another study conducted in Tanzania among midwives regarding competency on AMTSL it was reported that, there is high percentage of correct performance of three components of AMTSL which include timely administration of uterotonic drugs within one minute (74.3%), counter cord traction (94.3%), and uterine massage 15 min for the first 2 h (45.7%) [12]. This difference can be attributed to the smaller number of participants; only midwives were involved compared with the current study which had 340 participants and involved all health care providers working in labour ward and met the criteria.

HCPs aged 45 years and above were significantly associated with adequate skills related to AMTSL. This is in line with findings in the study conducted in Tanzania, in which HCPs who were aged 40 years and above had good skills in the prevention of postpartum haemorrhage using AMTSL [4]. The possible reason could be working experience in labor ward [4, 8].

Regarding health facilities, HCPs working in the health centers were less likely to have adequate practice on AMTSL. This finding is in line with findings from different studies, example study done in Ethiopia by Lami et al., who reported that those who were working in health center were less likely to be associated with practicing AMTSL [11]. This was also reported in the study conducted in Kenya [8].

## Limitation of the study

We used direct observation to assess provision of care on AMTSL. With direct observation the person observed could change the behaviour which could influence the finding. In order to reduce this, we used the inter-rater reliability measures during clinical observation and rating in order to reduce bias and strengthening the study.

## Conclusion

In conclusion, despite majority of HCPs having adequate knowledge on AMTSL, but they lacked skills. Sex, professional qualification and previous training (male) were found to have an influence on the levels of knowledge about AMTSL among HCPs. However, factors that influenced knowledge were contrary to those which influenced skills such as age and level of health facility. We recommend that there should be scheduled, timely and adequate on-job training, mentoring and supportive supervision on AMTSL among HCPs. The training should focus to nurses working in labor ward and their curriculum should be reviewed. Additionally, more researches in the nature of intervention that covers a large scale should be done to address issues of knowledge and skills on AMTSL.

## Data Availability

Data set is available upon request to the corresponding author.

## Abbreviations

AMTSL: Active Management of Third stage of Labour
BEmONC: Basic Emergency for Obstetric and Neonatal Care
CCT: Controlled Cord traction
CEmONC: Comprehensive Emergency for Obstetric and Neonatal Care
HCPs: Health care providers
JHPIEGO: John Hopkins Program for International Education of Gynaecological and Obstetric
MMR: Maternal Mortality Ratio
PPH: Postpartum Haemorrhage
